# Prevalence of sleep-related breathing disorders in children with malocclusion

**DOI:** 10.4317/jced.56855

**Published:** 2020-06-01

**Authors:** Ivette Vázquez-Casas, Oscar Sans-Capdevila, Jordi Moncunill-Mira, Alejandro Rivera-Baró

**Affiliations:** 1 Graduate in dentistry from University of Barcelona, Master of Ortodoncia y malformaciones dentofaciales from Sant Joan de Déu /University of Barcelona; 2 Medical director of AdSalutem Institute. Coordinator of Sleep disorders unit from Sant Joan de Déu Hospital; 3 Adjunct in Department of Odontopediatria and Orthodontics from Sant Joan de Déu Hospital. Hospital Dentistry, Clinical Orthodontics & Periodontal Medicine Research Group. Institut de Recerca Sant Joan de Déu (IRSJ), Fundació Sant Joan de Déu (FSJ); 4 Director in Department of Odontopediatria and Orthodontics from Sant Joan de Déu Hospital. Hospital Dentistry, Clinical Orthodontics & Periodontal Medicine Research Group. Institut de Recerca Sant Joan de Déu (IRSJ), Fundació Sant Joan de Déu (FSJ)

## Abstract

**Background:**

The paediatric population has a high incidence of sleep-related breathing disorders (SRBD). A notable risk factor is the presence of craniofacial abnormalities. The objective of the study was therefore to survey the prevalence of SRBD in patients presenting for interceptive treatment.

**Material and Methods:**

Prospective study with a sample of 249 healthy patients. The “Paediatric Sleep Questionnaire” and “Sleep Disturbance Scale for Children” were completed by the children’s parents and the results were evaluated. All patients had their medical records reviewed and underwent orthodontic diagnosis by oral examination, as well as dental cast and cephalometric analysis. Finally, we compared the results of the pre- and post-treatment questionnaires of 50 patients in the sample.

**Results:**

Based on the results of the questionnaires, 22.8% of the study sample had SRBD. No statistically significant correlation was found between SRBD and the anthropometric characteristics and occlusal variables assessed. According to the cast analysis, patients with SRBD had a smaller maxillary width (*p*<0.003), and according to the cephalometric study, less overbite (*p*<0.003). Furthermore, the prevalence of SRBD was higher among patients with a history of adenotonsillectomy (*p*<0.02). Comparison of the results of pre- and post-treatment questionnaires revealed significant differences after orthodontic treatment (*p*<0.0005).

**Conclusions:**

It is necessary to identify the presence of SRBD in orthodontic patients given its high prevalence. Patients with SRBD have a smaller maxillary width and less overbite.

** Key words:**Sleep-related breathing disorders, paediatric sleep questionnaire, cephalometry.

## Introduction

Sleep-related breathing disorders are a syndrome of upper airway dysfunction characterised by the presence of snoring and/or respiratory effort secondary to an increase in airway resistance and pharyngeal obstruction. They make up the second category in the International Classification of Sleep Disorders (ICSD-3, 2015) and comprise a broad spectrum of clinical entities with a gradient of severity: Obstructive Sleep Apnoea Syndrome (OSAS) is the most severe such disorder and affects 1-3% of the paediatric population.

Left untreated, SRBD can lead to serious complications such as growth retardation, neurocognitive and behavioural disorders, attention-deficit/hyperactivity disorder, cardiovascular problems and metabolic disorders.

The main risk factors associated with paediatric age are adenotonsillar hypertrophy and craniofacial anomalies. Craniofacial development is influenced by genetics and functional factors. Meanwhile, mouth breathing causes an increase in nasal respiratory resistance, altering muscular balance and affecting craniofacial development in the child, thus increasing the risk of malocclusion.

The gold standard for diagnosis is polysomnography, but due to the complexity of the procedure and its high cost, questionnaires have been developed as a screening method. The Pediatric Sleep Questionnaire (PSQ) and the Sleep Disturbance Scale for Children (SDSC) are among the most widely used in the paediatric population.

The PSQ, which was developed by Chervin (1) in 2007 for the diagnosis of sleep-related breathing disorders, has a high sensitivity and diagnostic specificity (0.78 and 0.72, respectively). A validated Spanish translation of the PSQ by Vila *et al.* (2), who obtained a concordance of 91% for the diagnosis of polysomnography, is available. The questionnaire was also validated by a study conducted by Bertran *et al.* (3) in 2015, which reported a sensitivity of 0.714 and specificity of 0.521.

As for the Sleep Disturbance Scale for Children (SDSC), it was developed by Bruni (4) in 1996 for the diagnosis of all sleep disorders (sensitivity of 0.89 and specificity of 0.74), the score correctly identifying 73.4% of the control group and 89.1% of SRBD subjects.

It was therefore decided to carry out this study with the following objectives:

- Determine the prevalence of sleep-related breathing disorders (SRBD) using questionnaires in patients presenting to Sant Joan de Déu Hospital for interceptive orthodontic treatment.

- Assess the correlation between the onset of SRBD and dentofacial characteristics.

- Compare the results of pre- vs. post-orthodontic treatment questionnaires.

## Material and Methods

The protocol was approved by the ethics committee of Sant Joan de Déu Hospital in Barcelona under number PIC-84-17.

All the patients who presented to the orthodontic department of Sant Joan de Déu Hospital and required interceptive treatment from April 2016 to December 2017 were consecutively enrolled. Patients with craniofacial malformations and/or respiratory and neurological disease were excluded from the study 

The final study sample consisted of 249 patients. Two sleep questionnaires and anthropometric characteristics were reviewed for each patient, and a comprehensive orthodontic assessment was carried out by oral examination and by dental cast and cephalometric analysis.

Sleep questionnaires 

The questionnaires were completed by the parents and/or legal guardians of the patients before orthodontic treatment. Questionnaires were also filled in at the end of the study for 50 patients from the sample who completed treatment during the study period. The questionnaires used were:

- Pediatric Sleep Questionnaire (PSQ): a 22-item questionnaire which analyses the presence of respiratory symptoms, enuresis, excessive sleepiness, headache and symptoms of hyperactivity and attention deficit. The possible answers are Yes, No and Don’t know. The total score was calculated by dividing the number of affirmative answers by the total number of questions. The validated cut-off was 0.33.

- Sleep disturbance scale for children (SDSC): a 26-item inventory rated on a 5-point Likert-type scale, with higher scores representing greater symptom severity. It includes six subdomains related to sleep: disorders of initiating and maintaining sleep, sleep breathing disorders, arousal disorders, sleep-wake transition disorders, excessive daytime sleepiness, and sleep hyperhidrosis. The total sleep score ranges from 26 to 130 points, with a cut-off score of 39. Of all the areas assessed with the rating scale, the most relevant to our study was the respiratory domain (SDSC.R), which was therefore reviewed separately using items 13, 14 and 15. The study sample was divided into three groups according to the risk of SRBD: high (12-15 points), moderate (7-11 points) and low risk (3-6 points).

Parents were also asked whether their child had undergone adenotonsillectomy prior to orthodontic treatment.

Anthropometric variables

The height and weight of each patient were recorded to calculate the body mass index (BMI) and assess the correlation with the presence of sleep-related breathing disorders. The patients were classified according to the nutritional diagnosis, taking into account the BMI z-score:

- Healthy weight: Percentile >=5 and <85.

- Underweight: Percentile <5.

- Overweight: Percentile >=85 and <95.

- Obesity: Percentile >=95.

Oral examination 

Angle’s classification was used to classify molar occlusion as Class I, II or III, differentiating between right and left.

Patients were examined for the presence or absence of cross-bite, which describes a malocclusion in which a mandibular tooth or teeth have a more buccal or labial position than the antagonist maxillary tooth.

Patients were examined for the presence or absence of open bite, which describes a malocclusion with an overbite of less than 0 mm.

Model analysis

**Figure 1:**
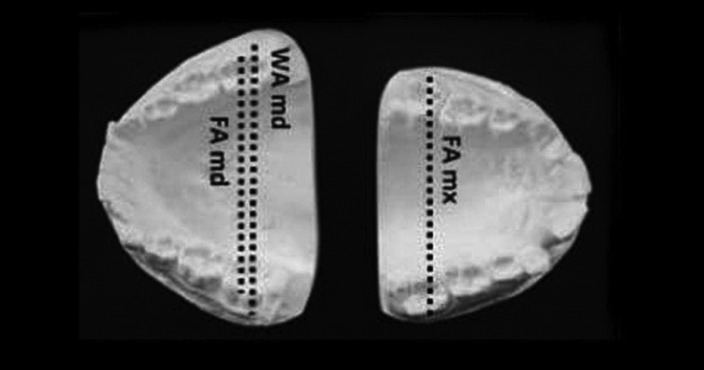
Measurements performed on dental casts according to the WALA Ridge analysis. We measured the distance between the crown centres of the maxillary and mandibular arches (mx FA, md FA) relative to the mandibular WALA ridge (md WA).

WALA Ridge analysis was used to evaluate the cross-sectional measurements of the patients (Fig. 1). The measurements were taken using a manual calliper on the dental casts made prior to orthodontic treatment, measuring the distance between crown centres (FA) in the maxillary and mandibular arch relative to the mandibular WALA ridge (md WA). Mandibular molar compensation was taken into account to quantify the degree of compression and necessary expansion. Four variables were thus obtained for the statistical analysis: md WA, md FA, mx FA and Expansion.

-Cephalometric analysis

**Figure 2:**
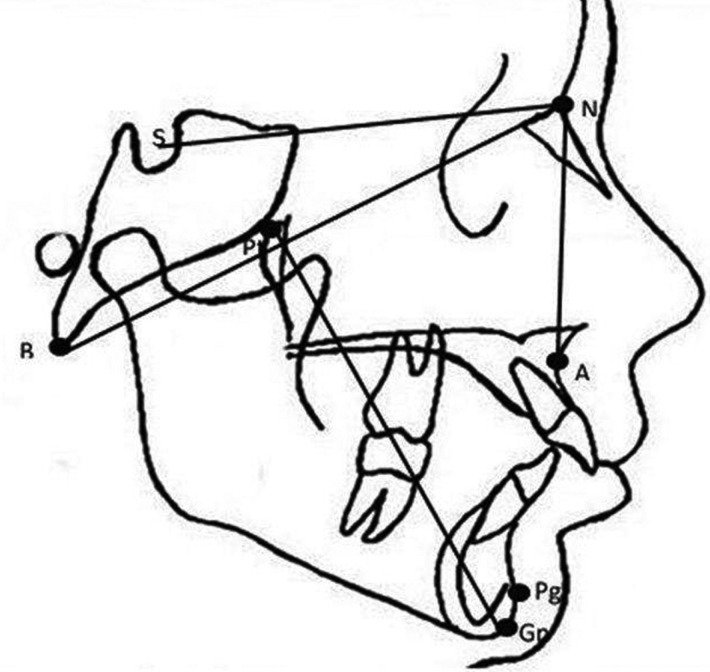
Cephalometric points used: A: most posterior point of the anterior curvature of the maxilla; Nasion (N): most anterior point of the frontonasal suture; Pogonion (Pg): most anterior point of the mandibular symphysis; Basion (Ba): most anteroinferior point of the foramen magnum; Pterygoid (Pt): most posterosuperior point of the pterygomaxillary fossa; Gnathion (Gn): point formed by the intersection of the facial and mandibular planes; Sella (S): saddle-shaped depression in the sphenoid bone. Lines: APg dental plane; NPg facial plane; Ba-N basicranial plane; Pt-Gn facial axis.

The data were complemented by a cephalometric study of patients prior to orthodontic treatment. All teleradiology images were obtained according to a standardised protocol and using the same X-ray equipment. Cephalometric analysis was performed using Nemoceph software. The plot was traced using a series of points and planes to perform linear and angular measurements, as shown in Figure 2. The measures studied are part of the Steiner and Ricketts analysis:

- Overjet, the horizontal distance between the upper incisal edge and the vestibular side of the lower incisors.

- Overbite, the vertical distance between the upper and lower incisal edge.

- Facial axis, the postero-inferior angle formed by the basicranial plane (Ba-Na) and the facial axis (Pt-Gn). Describes the general growth of the face. The value measured made it possible to determine the growth pattern of the patients, classifying them as mesofacial, brachyfacial and dolichofacial.

- ANB, the angle formed by the Nasion-point A and Nasion-point B planes. Defines the anteroposterior discrepancy between the maxilla and mandible, indicating the skeletal class.

- SNA, the angle formed by the sella-Nasion plane and Nasion-point A. Indicates the anteroposterior location of the maxilla with respect to the base of the skull.

- SNB, the angle formed by the sella-Nasion plane and Nasion-point B. Indicates the anteroposterior location of the mandible in relation to the base of the skull.

Statistical analysis

A descriptive statistical analysis of the data was performed. Comparisons were made between positive and negative sleep questionnaires using the values obtained in the oral examination and the cast and cephalometric analysis using the Chi-square test for qualitative variables and the Student’s t test for quantitative variables. ANOVA was used to assess variables with more than two categories.

The SPSS program (IBM Corp. Armonk, NY) was used for statistical analysis of the data obtained. A *p* value <0.05 was considered statistically significant.

## Results

The final sample consisted of 249 patients, 123 girls and 126 boys. The average age was 8.52 +/-1.30 years.

After calculating the scores of the questionnaires, an average prevalence of SRBD of 22.8% (56.3 patients) was obtained for the study sample (Table 1).

**Table 1:**
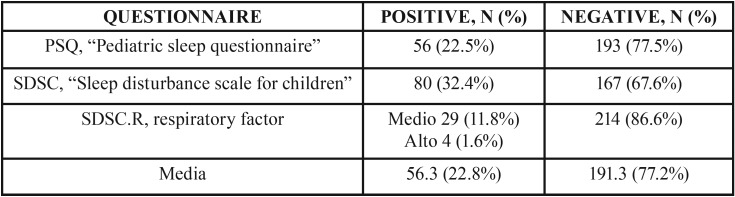
Results of questionnaires at T1.

The Kappa index was used to assess the concordance between questionnaires, showing moderate agreement (0.479), so the correlation between the results of the questionnaires and dentofacial characteristics was carried out separately with the different questionnaires.

A total of 15% of the sample had previously undergone surgery for adenoidectomy or tonsillectomy or both at the same time. The results of the questionnaires were analysed according to the patient’s history of surgery, showing a significantly higher incidence of SRBD in previously operated vs. non-operated patients.

Meanwhile, 69% of the sample had a normal BMI and analysis of the relationship between nutritional status and the presence of SRBB showed no statistically significant differences.

The results of the questionnaires revealed no statistically significant differences with regard to molar occlusion. It was found that 63.5% of the sample had a crossbite, with no statistically significant differences observed between questionnaire results. Furthermore, 24.5% had an open bite, with no differences observed between questionnaire results.

In the cross-model analysis ( Table 2, Table 3), the only statistically significant difference observed was maxillary width (mx FA) according to the SDSC.R (*p* <0.00); in the PSQ, the value approached significance (*p* < 0.06), with a smaller maxillary width in the group with respiratory sleep disorders.

**Table 2:**
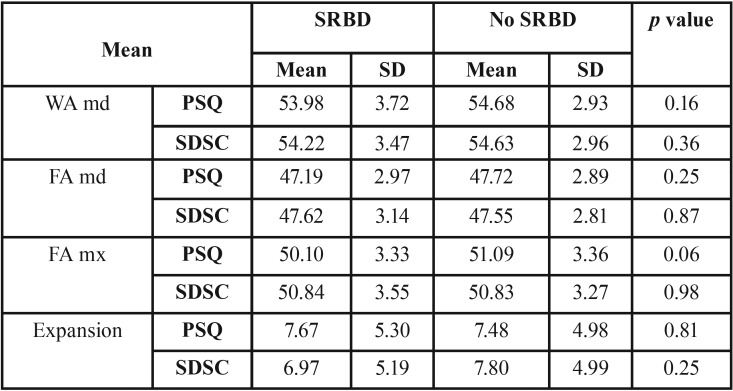
Mean and standard deviation (SD) of measurements assessed in the cross-model analysis of the entire sample according to the results of the PSQ and SDSC questionnaires.

**Table 3:**
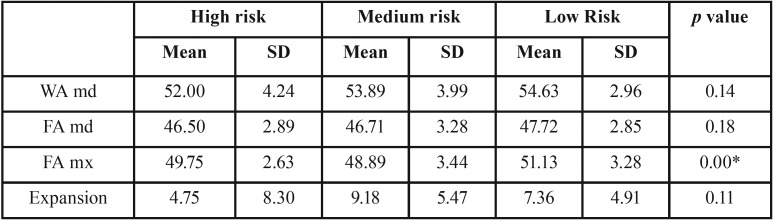
Mean and standard deviation (SD) of measurements assessed in the cross-model analysis of the entire sample according to the results of the SDSC.R.

The correlation between questionnaire results and skeletal discrepancy in the patients was also assessed, with no statistically significant relationship observed for any of the variables. The same results were obtained when assessing the growth pattern.

Overjet in the patients was contrasted with questionnaire results, showing no statistically significant differences. However, according to the SDSC.R, statistically significant differences were observed for overbite, which decreased as the risk of SRBD increased.

Comparison of the questionnaire results of 50 patients before and after orthodontic treatment showed no changes for PSQ, unlike for SDSC and SDSC.R.

## Discussion

The prevalence of SRBS varies depending on the sample selected and the diagnostic criteria used. The prevalence found in our study was 22.8%, slightly higher than in previous studies (5,6). This may be due to the fact that all the patients had malocclusion and that questionnaires were used as the diagnostic method.

Adenotonsillectomy is widely described in the literature as the first line of treatment for Obstructive Sleep Apnoea Syndrome in children, curing between 25-75% of patients depending on the study (7). Even so, patients who had undergone the procedure had more symptoms of SRBD, which is consistent with previous studies (8).

With regard to BMI, our study found that obesity is not a risk factor for OSAS in children, unlike in adults (9,10).

Craniofacial development is influenced by genetic and functional factors: sleep-related breathing disorders may thus lead to abnormal development of the oral and nasal cavity, altering its structure as well as the position of the tongue, and increasing the risk of malocclusion (11). The main objective of orthodontic analysis was therefore to assess the correlation between SRBD and the presence of malocclusion.

Oral examination was used to assess molar occlusion, with no statistically significant relationship identified. This is consistent with the findings of Carvalho (12) and Thome (13). The next variable analysed was the presence of open bite, with no differences found between groups, as in the studies by Ikavälko (5) and *Pi*rila-Parkinnen (8). In contrast, Carvalho *et al*. (12), who compared 10 healthy patients vs. 40 subjects with SRBD diagnosed by polysomnography, found significant differences between groups with regard to the presence of open bite.

As regards the presence of crossbite, while it has been correlated with respiratory disorders (9,12,14), our study found no statistically significant differences. In contrast, maxillary width was found to be significantly smaller in patients with SRBD, with a difference of 2.2 mm between patients with a low vs. moderate risk of SRBD. This finding was consistent with those of the studies reviewed (8,9).

It has been widely described that patients with SRBD have retrognathia or mandibular micrognathia, with a tendency toward Class II malocclusion (15). This is usually assessed using the ANB angle from Steiner’s analysis, and increased ANB values have been reported in the literature in patients with OSAS vs. controls, although the difference between patients with vs. without SRBD obtained in the systematic review by Katyal *et al.* was less than 2º, which is not clinically relevant (7).

According to the literature, there is a correlation between mouth breathing and increased tendency toward vertical growth of the face (15,16). Our study found no statistically significant differences, although a tendency toward dolichofacial growth patterns was observed in patients with SRBD.

As discussed above, in patients where SRBD was associated with Class II malocclusion and open bite, this was usually accompanied by greater overjet and less overbite (8,14). Although greater overjet and less overbite were observed in our study in patients with positive questionnaire results, significant differences were only observed for the latter association.

When comparing the results of the questionnaires between T1 and T2, it should be noted that only 50 patients completed treatment during the study period. While a significant improvement was observed in questionnaire results at the end of the treatment, due to the small sample size, the difference was not statistically significant. Future studies will include the questionnaire data of the entire study sample at T2 and assess if there is relationship with the type of treatment carried out.

## Conclusions

1. It is necessary to identify the presence of SRBD in orthodontic patients, given its high prevalence.

2. Patients with SRBD have a smaller maxillary width and less overbite.

3. Larger studies are needed to determine the influence of orthodontic treatment in patients with sleep-related breathing disorders.
